# Concurrent prediction of in-hospital mortality and length of stay using single-task, multiclass and multitask machine learning

**DOI:** 10.1136/bmjdhai-2025-000071

**Published:** 2025-12-16

**Authors:** Jia Wei, Jo Gay, Andrew Brent, David Clifton, Sarah Walker, David Eyre

**Affiliations:** 1 Nuffield Department of Medicine, University of Oxford, Oxford, UK; 2 Oxford University Hospitals NHS Foundation Trust, Oxford, UK; 3 Department of Engineering Science, University of Oxford, Oxford, UK; 4 Nuffield Department of Population Health, University of Oxford, Oxford, UK; 5 Big Data Institute, University of Oxford, Oxford, UK

**Keywords:** Artificial intelligence, Electronic Health Records, Machine Learning, Patient Care

## Abstract

**Objectives:**

Accurate predictions of discharge timing and in-hospital mortality could improve hospital efficiency, but clinician estimates are often inconsistent and imprecise. We evaluated if machine learning models could concurrently predict in-hospital mortality and length of stay (LoS) more reliably.

**Methods and analysis:**

We used electronic healthcare data from 1 November 2021 to 31 October 2024 from Oxfordshire, UK, using 2 years’ data for training and evaluating models using the final year’s data. The performance of task-specific extreme gradient boosting (XGB), logistic regression (LR), multilayer perceptron (MLP) and TabNet models for two tasks, mortality prediction and LoS prediction, was compared with a single multiclass XGB model predicting combinations of LoS and mortality and MLP and TabNet-based multitask learning model predicting both outcomes simultaneously. Predictions from the best-performing models were compared with discharge predictions made by clinicians.

**Results:**

Clinicians provided relevant discharge predictions for only 3%–5% of admissions, mostly close to discharge. Task-specific XGB models achieved an area under the receiver operating curve of 0.92 and 0.92 for predicting mortality and 0.83 and 0.72 for predicting LoS quartiles in elective and emergency admissions, respectively, outperforming task-specific LR, MLP and TabNet models. Neither the multiclass XGB nor the MLP or TabNet-based multitask models, predicting both outcomes simultaneously, consistently improved performance. The best-performing task-specific XGB models matched clinician LoS prediction accuracy in elective admissions and significantly outperformed clinicians in emergency admissions (p <0.001).

**Conclusion:**

Machine learning models can predict in-hospital mortality and LoS as well or better than clinicians, especially for emergency admissions. These models have the potential to be implemented in hospital settings, providing consistent predictions and thus enhancing discharge planning and hospital resource management.

**WHAT IS ALREADY KNOWN ON THIS TOPIC**

- Various machine learning models have been used to predict in-hospital mortality and length of stay (LoS), but most studies have analysed them separately and often just in specific hospital settings such as intensive care. Furthermore, few studies have directly compared model performance against clinician predictions.

**WHAT THIS STUDY ADDS**

- This study integrates diverse electronic healthcare features to develop task-specific models for mortality and LoS quartile prediction in a general hospital population, with extreme gradient boosting outperforming other approaches.
- Multitask learning approach offered limited performance gains.
- The best-performing models outperformed clinicians in predicting LoS for emergency admissions, where clinician predictions were often sparse and overly optimistic.

**HOW THIS STUDY MIGHT AFFECT RESEARCH, PRACTICE OR POLICY**

- Integrating automated LoS and mortality predictions in clinical settings using machine learning could lead to better discharge planning, resource allocation and patient flow management.

## Introduction

Increasing demand for healthcare services has placed significant pressure on limited available resources, highlighting the need to enhance hospital efficiency and capacity management.^1^ Discharge planning is a critical component of operational management, and early discharge planning helps reduce length of stay (LOS) and readmissions. Some hospitals have implemented discharge planning interventions, asking healthcare professionals to predict the date of discharge.^2–4^ However, there is a lack of evidence examining the accuracy of these predictions, which may also be resource intensive to generate and inconsistently performed.^5^ Identifying patients at risk of in-hospital mortality is important to bed-state predictions and also has potential to improve patient outcomes.

Several machine learning models using electronic healthcare (EHR) data have been developed to predict either mortality^6–10^ or LoS^11–16^ among hospitalised patients, with variable performance. However, these studies typically focus on a single prediction task, overlooking the interconnected nature of real-world clinical outcomes. Both discharge and mortality may lead to a bed becoming available, but these events are preceded by different health trajectories, with models having to track recovery or deterioration respectively. A few studies have explored predicting both mortality and LoS using deep learning models, but these tasks were trained separately.^17 18^ Multitask learning, a technique that enables simultaneous prediction of multiple related outcomes by sharing feature representations between tasks,^19^ offers a promising alternative. The approach has shown success in various healthcare applications, for example, cancer prognosis,^20^ postoperative complications,^21^ echocardiogram interpretation,^22^ mental disorder diagnosis^23^ and Alzheimer’s disease diagnosis.^24–26^ One study used a neural network-based multitask learning model to predict inpatient flow and LoS with improved accuracy compared with single-task models, although it did not address mortality.^27^ A recent study introduced a multimodal multitask learning framework to perform LoS regression and mortality classification tasks simultaneously using the Medical Information Mart for Intensive Care database, achieving better performance than conventional methods.^28^ However, there was limited evaluation of the practical application of this model, and no comparison was made with clinician predictions.

In this study, we first examined the accuracy of clinician-provided discharge estimates from a large UK teaching hospital group. We then built machine learning models to predict inpatient mortality and LoS quartiles, comparing the performance and computational efficiency of separate task-specific models for mortality and LoS, with a multiclass model predicting combinations of mortality and LoS together and a multitask learning model predicting both mortality and LoS simultaneously, but as separate outcomes. Last, we assessed the models’ predictions against those of clinicians. We hypothesised that machine learning models could predict inpatient mortality and LoS with higher accuracy and consistency than clinician estimates, and a multitask learning model would outperform separate models by capturing shared information between related clinical outcomes.

Our study makes novel contributions in: (1) using comprehensive EHR data from all hospital inpatients rather than just a specific population like intensive care; (2) building predictive models using rich EHR-derived features to jointly predict mortality and LoS quartiles, rather than the dominant previous approach of predicting each task separately; and (3) systematically evaluating clinician-provided discharge estimates and comparing model predictions against clinician estimates to assess real-world applicability, which has been rarely examined in prior work.

## Methods

### Data extraction

We used data from the Infections in Oxfordshire Research Database (IORD), which includes de-identified electronic health records from Oxford University Hospitals, four teaching hospitals serving around 1% of the UK population. IORD has approvals from the National Research Ethics Service South Central-Oxford C Research Ethics Committee (19/SC/0403), Health Research Authority and Confidentiality Advisory Group (19/CAG/0144) as a de-identified database without individual consent.

We extracted data on all adult inpatients (≥16 years) between 1 November 2021 and 31 October 2024, the most recent and complete 3 years of data available at the time of extraction. Admissions were classified as elective (scheduled in advance) or emergency (details in online supplemental methods).

10.1136/bmjdhai-2025-000071.supp1 Supplementary data

Clinician discharge date predictions were made by individual clinicians or during multidisciplinary team meetings and recorded in the EHR. There was no formal guidance or standardised tool for making these predictions; they were based on clinician judgement, informed by the current clinical status, planned investigations, availability of post-discharge support services and experience. This process varied across clinical teams and specialties, reflecting local workflow practices and priorities. Not all patients had predictions documented, as this was not prioritised in all hospital areas.

### Features and preprocessing

To build prediction models, we used the same feature set and preprocessing as previously described.^29^ A total of 1152 features were created and grouped into 15 feature categories based on domain knowledge and previous literature (online supplemental methods), including index date-related features, demographics, comorbidities, current admission, ward stay, current diagnostic category, procedures, antibiotics prescriptions, medication, microbiology tests, radiology investigation, readmissions and previous hospital stay, hospital capacity factors reflecting crowdedness, vital signs and laboratory tests. Missing categorical features were imputed using the mode, while numerical features were imputed using the median.

### Prediction outcome

We predicted in-hospital mortality and future hospital LoS at an index date and time for all patients currently in the hospital. The in-hospital mortality was extracted as a binary event, and future hospital LoS was categorised into quartiles, reflecting an operationally relevant framework for distinguishing between short, moderate and prolonged stays, and to reduce class imbalance:

- Elective admissions: Q1: 0–1, Q2: 2–3, Q3: 4–10, Q4: >10 days.
- Emergency admissions: Q1: 0–2, Q2: 3–6, Q3: 7–14, Q4: >14 days.

Predictions were made daily at 12 noon to align with the end of typical hospital ward rounds. Each patient contributed once to the dataset each day during their hospital admission. Separate models were built for emergency and elective admissions as predictive features plausibly differ by the admission type. Models were trained using data from the first 2 years (1 November 2021 to 31 October 2023) and evaluated using data from the final year (1 November 2023 to 31 October 2024), which allowed us to assess model performance on separate held-out test data, including capturing the impact of temporal shifts in input features and discharge practice (online supplemental figure S1).

### Model architectures

In our single-task model analysis, we used binary extreme gradient boosting (XGB) to predict mortality and a separate multiclass XGB to predict hospital LoS (see online supplemental methods and online supplemental table S1 for details of hyperparameter tuning). We used 20% of the training dataset as validation data for feature selection, retaining the 200 most informative features based on this optimising performance in a previous study,^29^ calibrating the predicted probability from XGB models using isotonic regression and determining the best threshold for predicting mortality by optimising the F1 score (jointly maximising precision and recall). We compared the XGB models to logistic regression (LR), multilayer perceptron (MLP) and TabNet^30^ models, using the same 200 features selected for the XGB models (details in online supplemental methods).

We next fitted an XGB model similarly to above but combining mortality and LoS quartiles into a single multiclass model with seven outcome categories comprising linked mortality and LoS outcomes:

- Elective: died in hospital within 0–1 days, 2–3 days or 4–10 days; discharged alive within 0–1 days (Q1), 2–3 days (Q2) or 4–10 days (Q3); or remained in hospital >10 days (Q4).
- Emergency: died in hospital within 0–2 days, 3–6 days or 7–14 days; discharged alive within 0–2 days (Q1), 3–6 days (Q2) or 7–14 days (Q3); or remained in hospital >14 days (Q4).

Finally, we fitted multitask learning models^18 25^ using MLP and TabNet architectures using the same feature set as the single-task models to predict mortality and LoS quartiles simultaneously. The same loss functions and hyperparameters were used as in the single-task models. To properly combine the task-specific losses, we compared different loss weighting strategies, including uniform weighting (with different weights), random loss weighting^31^ and uncertainty weighting.^32^

### Performance assessment

Model predictions were evaluated using established metrics, comparing performance to actual events (online supplemental methods). Subgroup performance by demographics, time since admission and time to discharge was examined. We also compared the performance of the LoS models with clinician predictions. As clinician predictions were made at random timepoints throughout the day, for comparisons, a separate model prediction was made, using the time of the prediction, rather than 12 noon, as the index date and time for the data extract.

This study is reported adhering to the TRIPOD-AI guideline.^33^

### Patient and public involvement

Patients and the public have identified studying how healthcare is delivered and the experience of hospital inpatients as priorities through public engagement events and a dedicated public panel. A proposal outlining this specific study was reviewed by the IORD public panel and updated based on their feedback.

## Results

### Patient characteristics

From 1 November 2021 to 31 October 2024, a total of 37 574 adult elective admissions (31 306 patients) and 154 849 emergency admissions (94 702 patients) were included in the analyses. The median age was 65 (IQR: 46–79) years, 98 222 (51.0%) patients were female and 136 670 (71.0%) were recorded as white ethnicity (ethnicity missing in 43 214, 22.5%). Mortality rates were 0.4% (158/37 574) for elective and 4.3% (6629/154 849) for emergency admissions. The future LoS followed a skewed distribution, with medians of 2.1 (IQR: 1.2–4.6) days for elective and 2.2 (0.6–7.0) days for emergency admissions (online supplemental figure S2). Demographic characteristics were similar between the training and test datasets (online supplemental table S2).

### Clinician predictions

During the test period, clinicians made a total of 2117 and 14 334 predictions during 1265 (3.4%) elective and 7363 (4.8%) emergency admissions, respectively. Relevant predictions were made on 2081 (98.3%) and 13 946 (97.3%) occasions (the remainder had an anticipated discharge date earlier than the date that the prediction was made and was excluded). In elective admissions, most clinician predictions were made in a surgical subspecialty (41.6%) or a medical subspecialty (36.3%). In emergency admissions, most predictions were made in acute, emergency and geriatric medicine (35.6%), followed by a medical subspecialty (27.9%) and acute and general surgery (16.1%). The median (IQR) LoS for patients with clinician predictions was longer than in the overall dataset, being 3.5 (1.4–8.2) days for elective and 7.1 (3.1–14.5) days for emergency admissions (online supplemental table S2).

**Figure 1:**
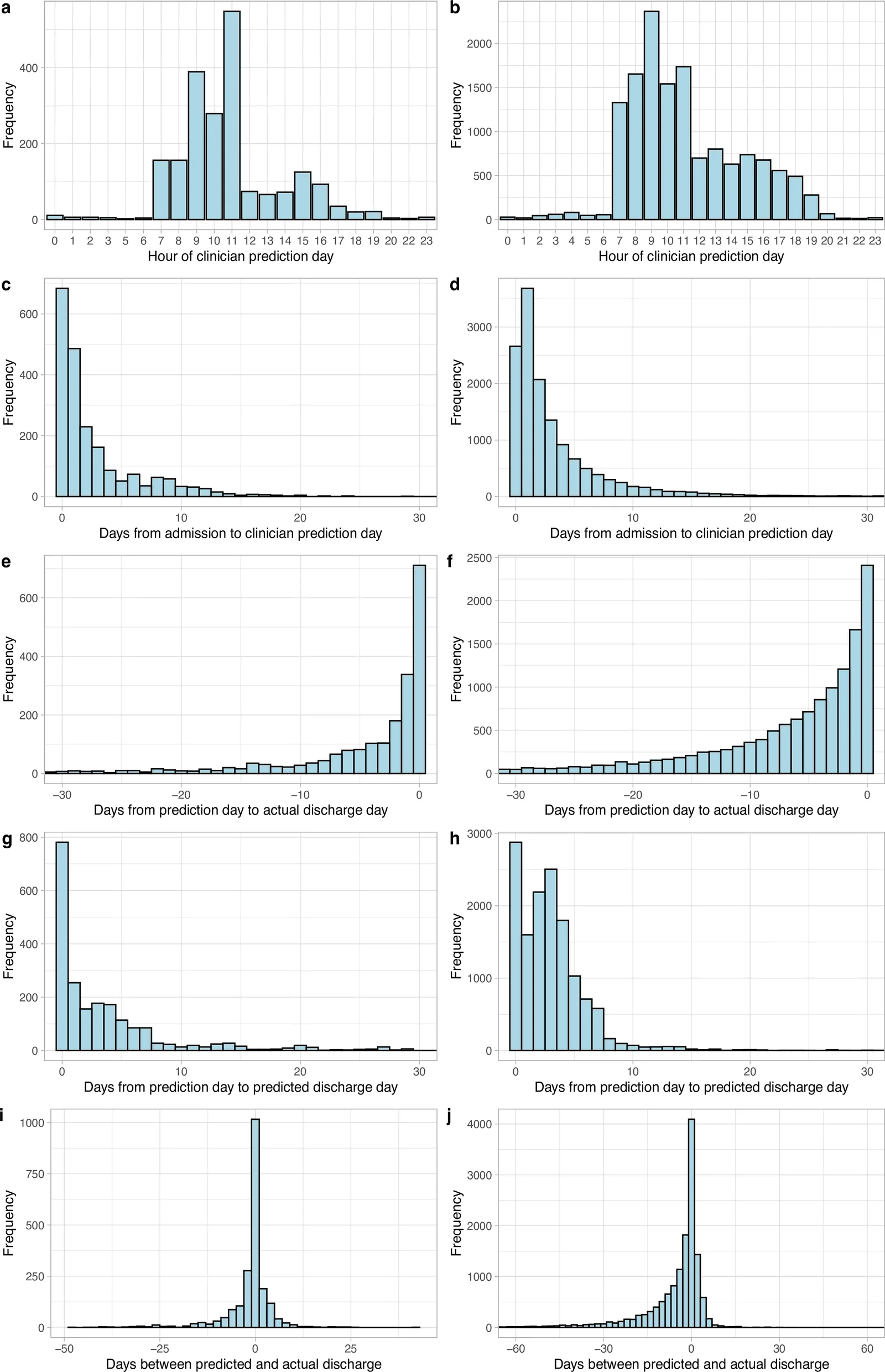
Clinician predictions of discharge. (a, b) Time of the day of clinician prediction. (c, d) Days from admission to when clinicians made predictions (prediction day). (e, f) Days from prediction day to actual discharge day. (g, h) Days from prediction day to predicted discharge day. (i, j) Days between clinician prediction and actual discharge. (a), (c), (e), (g) and (i) are elective admissions, while (b), (d), (f), (h) and (j) are emergency admissions.

Most clinician predictions were made in the morning between 9 and 11am for elective and 7 and 11am for emergency admissions (figure 1a,b). Predictions were typically made 1.2 (IQR 0.4–3.6) days and 1.9 (0.7–4.3) days from admission (figure 1c,d) and 1.4 (0.3–6.2) days and 4.3 (1.3–11.2) days before actual discharge, for elective and emergency admissions, respectively (figure 1e,f). The prediction horizon was short; clinicians tended to predict patients being discharged in 1.5 (0.5–4.5) days and 2.6 (0.6–3.7) days for elective and emergency admissions, respectively. 981 (47.1%) elective and 3744 (26.8%) emergency predictions were for discharge that day or the following day.

For elective admissions, 859 (41.2%) predictions identified the correct discharge day, 661 (31.8%) were earlier than the actual discharge date and 561 (27.0%) were later than the actual discharge date. For emergency admissions, 3038 (21.2%) were correct; 7518 (53.9%) were earlier than the actual discharge date, that is, tended to be overly optimistic; and 3390 (24.3%) were later than the actual discharge date (figure 1g,h). Overall, clinician predictions were made inconsistently and had suboptimal accuracy.

### Single-task model performance

Predicting discharge at 12 noon, 186 627 and 960 354 patient-days were included in the analyses for elective and emergency admissions respectively.

#### Mortality prediction

**Figure 2:**
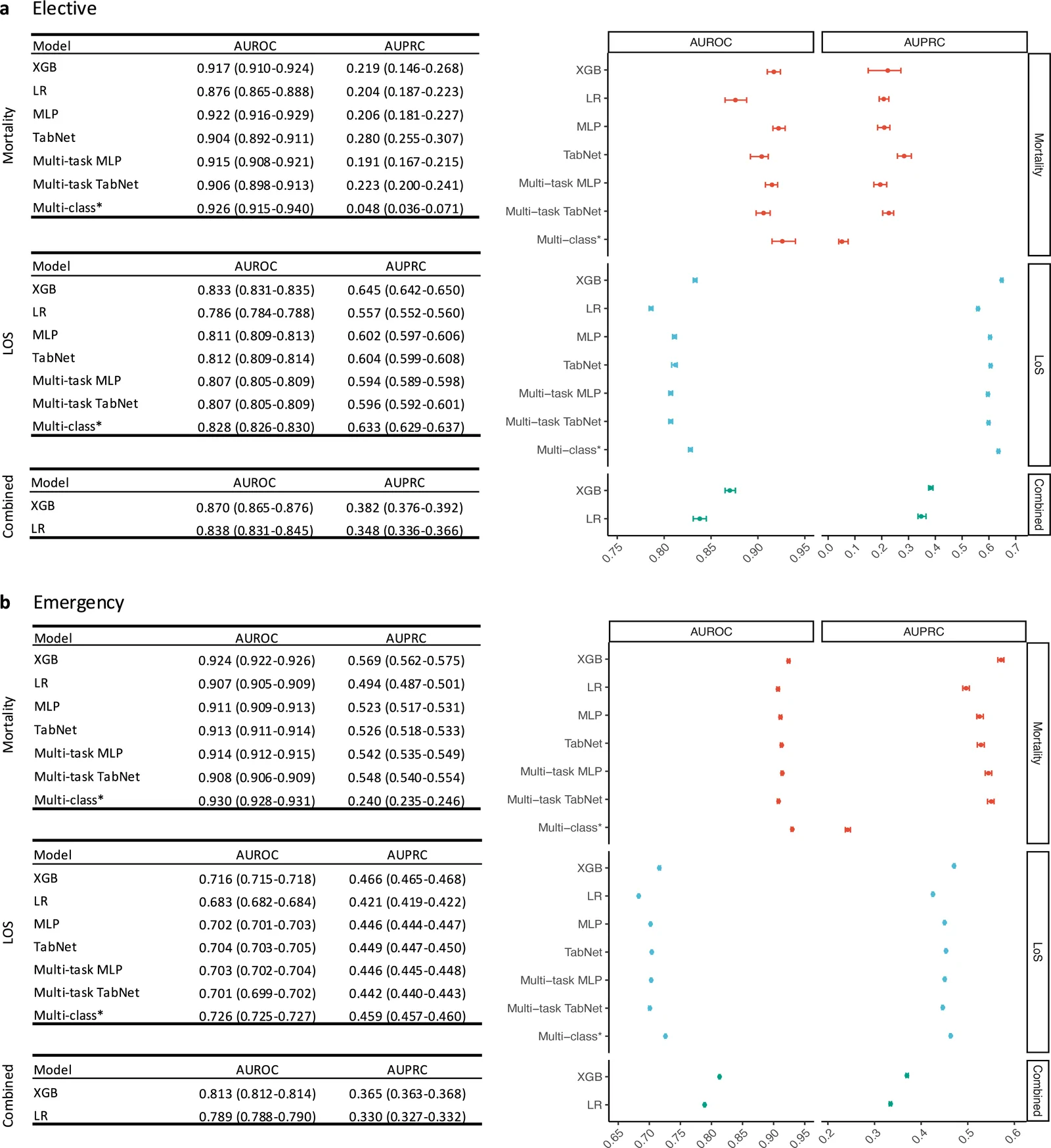
Comparison of model performance. Extreme gradient boosting (XGB), logistic regression (LR), multilayer perceptron (MLP), TabNet and multitask learning model were built for predicting mortality and future length of stay (LoS), and the multiclass XGB model was built for predicting combined mortality and LoS. *Macro-averaged performance for mortality classes (died in hospital) or LoS classes (Q1–Q4) from the multiclass XGB model predicting combined mortality and LoS. (a) Area under the receiver-operating curve (AUROC) and area under the precision-recall curve (AUPRC) for elective admissions. (b) AUROC and AUPRC for emergency admissions. Means with 95% CIs are calculated using bootstrap. In-hospital mortality occurred following 2982 (1.6%) elective and 79 646 (8.3%) emergency patient-days. Across all patient-days studied, the median future LoS was 3.2 (IQR 1.1–10.0) days and 5.9 (1.9–14.2) days for elective and emergency admissions, respectively (ie, longer than the overall LoS as longer admissions contributed proportionally more to the dataset than shorter admissions). In elective admissions, patient-days were distributed as 0–1 day (Q1, 20.1%), 2–3 days (Q2, 25.9%), 4–10 days (Q3, 29.0%) and >10 days (Q4, 25.0%). In emergency admissions, patient-days were distributed as 0–2 days (Q1, 25.8%), 3–6 days (Q2, 24.8%), 7–14 days (Q3, 23.8%) and >14 days (Q4, 25.6%).

For elective admissions, the XGB model achieved an area under the receiver operating curve (AUROC) of 0.917 (95% CI 0.910 to 0.924) and an area under the precision recall curve (AUPRC) of 0.219 (95% CI 0.146 to 0.268) for predicting inpatient mortality. Using a probability threshold that optimised F1 score in the validation dataset, the positive predictive value (PPV) was 0.262, negative predictive value (NPV) 0.989 and F1 score 0.242 (online supplemental table S3). The LR model yielded a lower AUROC of 0.876 (95% CI 0.865 to 0.888) and an AUPRC of 0.204 (95% CI 0.187 to 0.223), while the MLP model achieved a similar AUROC of 0.922 (95% CI 0.916 to 0.929) and an AUPRC of 0.206 (95% CI 0.181 to 0.227). The TabNet model had slightly lower AUROC of 0.904 (95% CI 0.892 to 0.911) but higher AUPRC of 0.280 (95% CI 0.255 to 0.307) (figure 2a) and provided the most consistent net benefit in a decision curve analysis between threshold probabilities of 5%–30% (online supplemental figure S3).

For emergency admissions, the XGB model achieved a similar AUROC at 0.924 (95% CI 0.922 to 0.926) and a higher AUPRC of 0.569 (95% CI 0.562 to 0.575) reflecting the higher mortality rate. At the optimal F1 threshold, the PPV was 0.532, NPV 0.963 and F1 score 0.551 (online supplemental tables S3 and S4). The XGB model was significantly better than the LR model (AUROC, 0.907 (95% CI 0.905 to 0.909); AUPRC, 0.494 (95% CI 0.487 to 0.501)) and also better than the MLP (AUROC, 0.911 (95% CI 0.909 to 0.913); AUPRC, 0.523 (95% CI 0.517 to 0.531)) and TabNet models (AUROC, 0.913 (95% CI 0.911 to 0.914); AUPRC, 0.526 (95% CI 0.518 to 0.533)) (figure 2b, online supplemental table S3). The XGB model yielded the highest net benefit across a broad range of thresholds (online supplemental figure S3).

**Figure 3:**
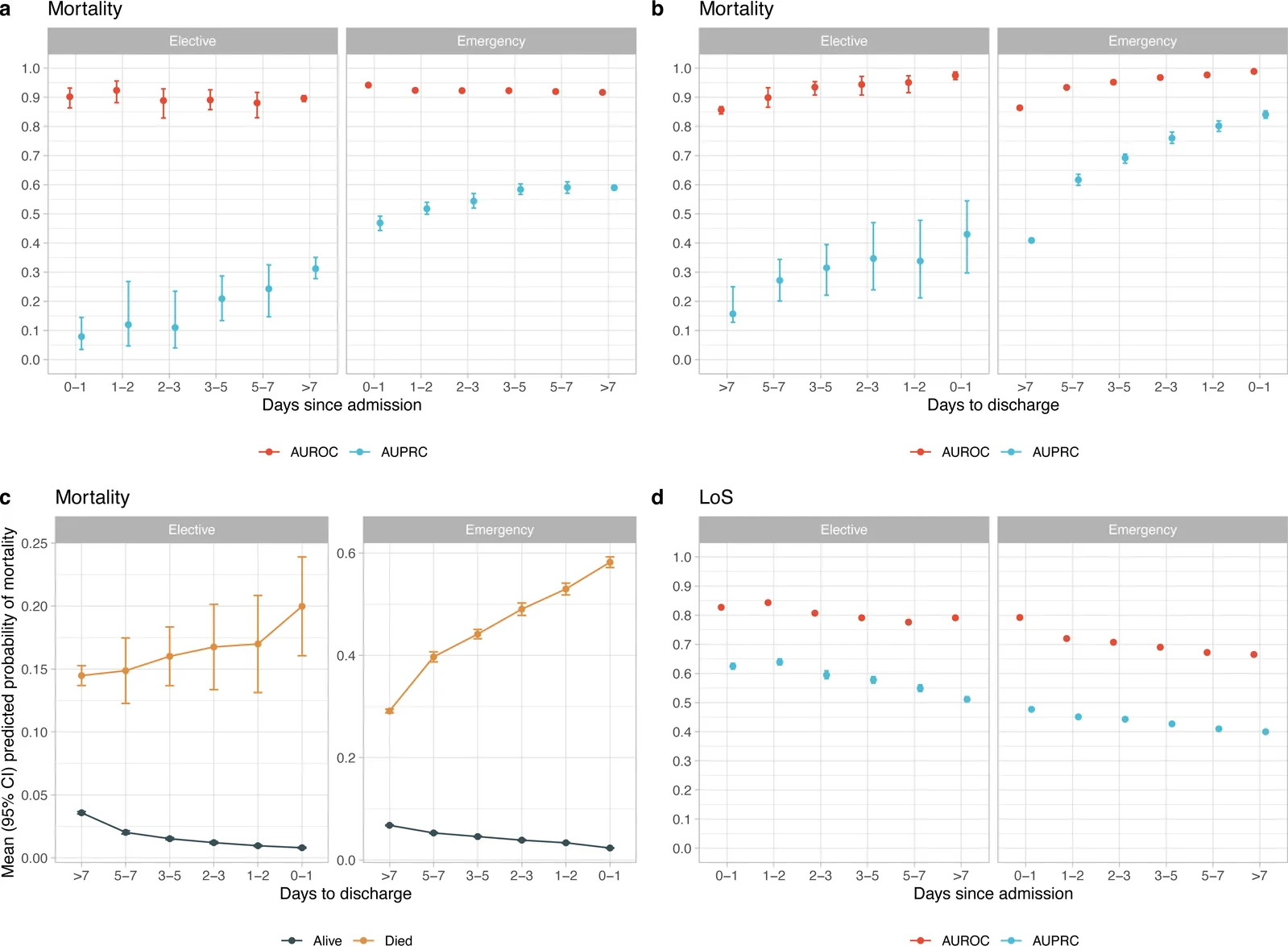
Changes in model performance and predicted probabilities by time in the test data using extreme gradient boosting models. (a) Changes in AUROC and AUPRC by time since admission for predicting mortality. (b) Changes in AUROC and AUPRC by time to discharge for predicting mortality. (c) Changes in predicted probability of mortality by time to discharge in patients who discharged alive and died in hospital (individual predicted trajectories are shown in online supplemental figure S4). (d) Changes in AUROC and AUPRC by time since admission for predicting length of stay (LoS). Mean with 95% CIs is calculated using bootstrapping. AUPRC, area under the precision recall curve; AUROC, area under the receiver operating curve.

For both elective and emergency admissions, AUROC remained consistent with varying time since admission, while AUPRC increased due to a higher mortality rate with longer LoS. Performance was higher when a prediction was made closer to discharge (figure 3a,b). Predicted probabilities of mortality increased for those who died the closer they were made to the date of death and decreased for those who were discharged alive when made closer to discharge (figure 3c, individual trajectories in online supplemental figure S4). Model performance was broadly similar despite some variations in age and admission speciality, and model fairness was achieved for most subgroups (online supplemental figure S5).

#### Future LoS prediction

The LoS prediction XGB model achieved a macro-averaged (giving the same weight to each LoS category or class) AUROC of 0.833 (95% CI 0.831 to 0.835), an AUPRC of 0.646 (95% CI 0.642 to 0.650) and an F1 score of 0.587 (95% CI 0.584 to 0.591) (online supplemental table S3). Q4 had the highest individual one-vs-rest AUROC (0.901), followed by Q1 (0.876), Q2 (0.788) and Q3 (0.768), that is, patients with the longest or shortest LoS were simplest to identify (online supplemental figure S6). LR, MLP and TabNet models performed slightly worse, with macro-averaged AUROCs of 0.786 (95% CI 0.784 to 0.788), 0.811 (95% CI 0.809 to 0.813) and 0.812 (95% CI 0.809 to 0.814), respectively (figure 2).

Performance was generally lower for emergency admissions compared with elective admissions, with the XGB model achieving a macro-averaged AUROC of 0.716 (95% CI 0.715 to 0.718) and an AUPRC of 0.466 (95% CI 0.465 to 0.468) and the highest individual AUROC in Q1 (0.813) and Q4 (0.760) (online supplemental figure S6). LR, MLP and TabNet models showed lower performance with AUROCs of 0.683 (95% CI 0.682 to 0.684), 0.702 (95% CI 0.701 to 0.703) and 0.704 (95% CI 0.703 to 0.705), respectively (figure 2, online supplemental tables S3 and S4).

AUROC was relatively stable by time since admission (around 0.80) for elective admissions, but decreased from 0.80 the day after admission to 0.65 >7 days since admission for emergency admissions, reflecting difficulty in predicting discharge for patients with longer hospital stays in whom discharge frequently depends on provision of social care, availability of which is not well captured in the available data (figure 3). Model performance varied slightly across age groups and specialties, but all subgroups achieved model fairness (online supplemental figure S5).

#### Feature importance

**Figure 4:**
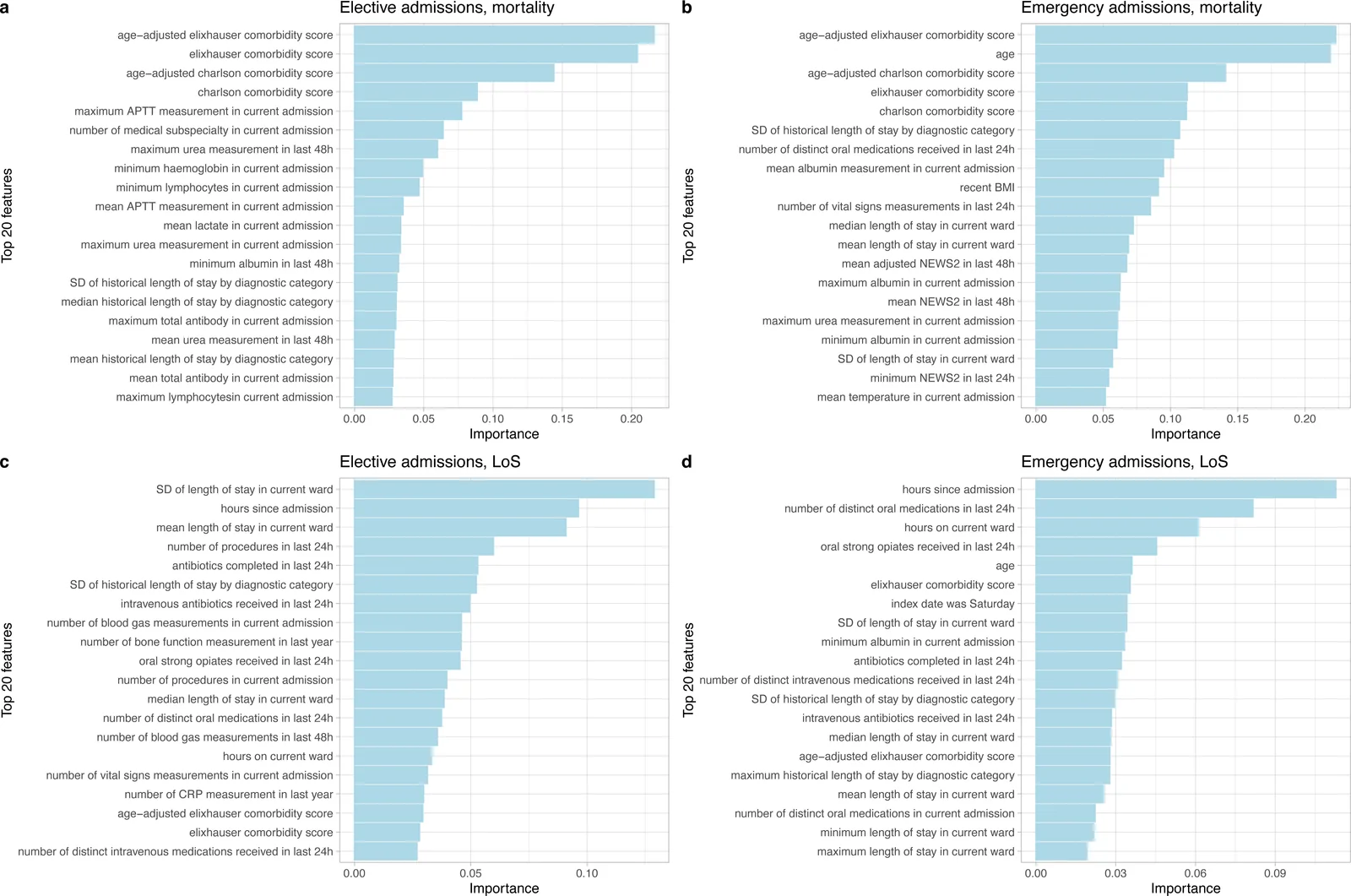
Feature importance from the extreme gradient boosting models predicting in-hospital mortality and future hospital length of stay (LoS) using SHapley Additive exPlanations values for elective and emergency admissions. The top 20 most predictive features are shown in the order of predictiveness. APTT, activated partial thromboplastin time; BMI, body mass index; CRP, C-reactive protein.

The top 20 features ranked by importance are shown in figure 4. The most predictive features for in-hospital mortality were Elixhauser and Charlson comorbidity scores, which were selected as the top 5 most important features for both elective and emergency admissions. Other important features were the number of specialties involved in the current admission to date and various laboratory test measurements (activated partial thromboplastin time, urea, haemoglobin, lymphocytes, lactate) for elective admissions and age, historical LoS, oral medications, albumin measurement and body mass index for emergency admissions.

Hours since admission, LoS on current ward, antibiotics and other medications were selected as the most important features predicting future LoS for both elective and emergency admissions.

### Combined multiclass model performance

Using a multiclass XGB model to predict combined mortality and LoS outcomes (online supplemental table S5), elective admissions had a macro-averaged AUROC of 0.870 (95%CI 0.865 to 0.876), with those dying in hospital 0–1 days later having the highest AUROC (0.960), followed by deaths in hospital 2–3 days later (0.924) and remaining in hospital for >10 days longer (0.898). The macro-averaged AUPRC was 0.381 (95% CI 0.376 to 0.392), with those dying in hospital in the next 0–10 days having low values <0.1 due to the low prevalence (online supplemental table S3 and figure S7). Macro-averaging the three mortality classes resulted in an AUROC of 0.926 (95% CI 0.915 to 0.940) (cf. separate binary XGB mortality model: 0.917 (95% CI 0.910 to 0.924) and LoS classes (discharged Q1–Q4) 0.828 (95% CI 0.826 to 0.830) (cf. separate XGB LoS model: 0.833 (95% CI 0.831 to 0.835) (figure 2).

For emergency admissions, the macro-averaged AUROC was 0.813 (95% CI 0.812 to 0.814) and the AUPRC 0.365 (95% CI 0.363 to 0.368), with the highest AUROC observed for admissions with mortality outcomes (deaths in 0–2, 3–6 and 7–14 days: 0.966, 0.929, 0.894) (online supplemental table S3 and figure S7). Macro-averaging mortality classes resulted in an AUROC of 0.930 (95% CI 0.928 to 0.931) (cf. separate model: 0.924 (95% CI 0.922 to 0.926)) and LoS classes 0.726 (95% CI 0.725 to 0.727) (cf. separate model 0.716 (95% CI 0.715 to 0.718)) (figure 2).

### Multitask model performance

We trained a multitask MLP model using the same feature set selected from single-task XGB models. Comparing different weighting strategies, the performance for mortality prediction was relatively stable, while higher performance was achieved for LoS prediction when a higher weight was assigned (online supplemental table S6). Therefore, the final models used a uniform weight of 1:10 for mortality:LoS.

For elective admissions, the multitask MLP model achieved an AUROC of 0.915 (95% CI 0.908 to 0.921) for mortality (vs 0.917 (95%CI 0.910 to 0.924) in separate XBG models) and 0.807 (95% CI 0.805 to 0.813) for LoS prediction in the test data (vs 0.833 (95% CI 0.831 to 0.835)). For emergency admissions, AUROCs were 0.914 (95% CI 0.912 to 0.915) for mortality (vs 0.924 (95% CI 0.922 to 0.926)) and 0.703 (95% CI 0.702 to 0.704) for LoS (vs 0.716 (95% CI 0.715 to 0.718)). Hence, while mortality performance was comparable to single-task XGB or MLP models, LoS performance was lower than XGB but comparable to MLP. A multitask TabNet model yielded similar performance to the multitask MLP model or single-task TabNet models (online supplemental table S3, figure 2).

### Model training times

To compare training times across models, we calculated the total time for hyperparameter tuning, model fitting and prediction. To ensure a fair comparison, 30 optimisation trials were used for each model, and the terminator improvement plot was examined to ensure the number of trials was sufficient. For elective admissions, there was a time saving associated with the combined models; the training time for the multitask MLP model and the multiclass XGB model was shorter than the combined training time for the separate mortality and LoS single-task XGB or MLP models. For emergency admissions, the multitask MLP model was faster than the combined mortality and LoS single-task MLP models but was slower than the sum of the time taken for the two single-task XGB models or for the multiclass XGB model. TabNet architectures required longer training time than other models (online supplemental figure S8).

### Comparison with clinician predictions

Using the best-performing single-task XGB model to predict future LoS at the same time point as clinicians had made a prediction, the model performance vs the actual discharge date resulted in a macro-averaged AUROC and AUPRC of 0.826 and 0.648 for elective admissions and 0.711 and 0.492 for emergency admissions. The model achieved similar accuracy to clinicians for elective admissions (0.566 vs 0.585, p=0.14 using McNemar’s test), but significantly outperformed clinicians for emergency admissions (0.442 vs 0.402, p<0.001).

**Figure 5:**
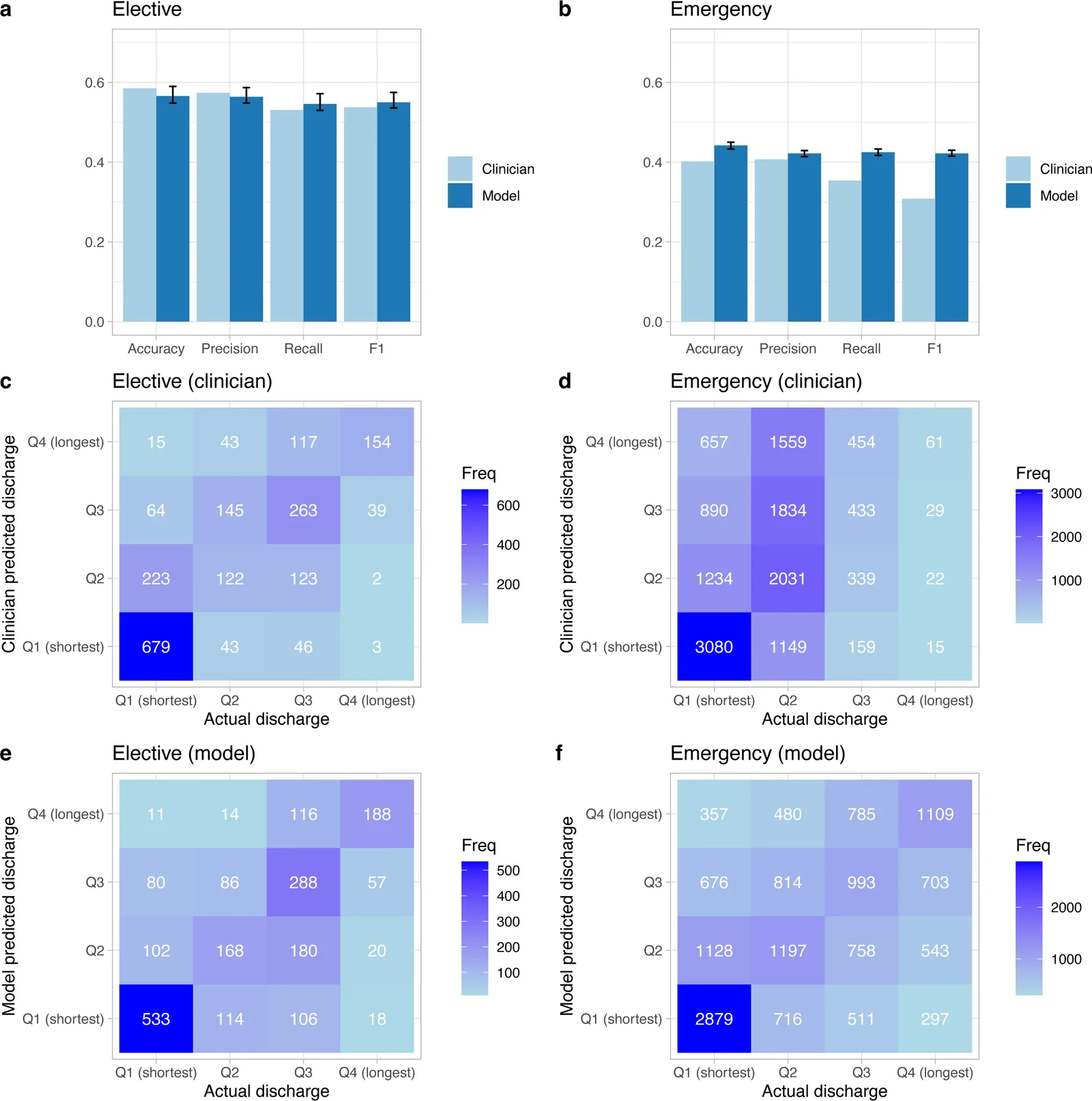
Comparison between clinician predictions and model predictions on future hospital length of stay (LoS) in the test dataset where clinicians made predictions. (a) Comparison of accuracy, precision, recall and F1 score between clinician and the best-performing extreme gradient boosting (XGB) model for elective admissions. (b) Comparison of accuracy, precision, recall and F1 score between clinician and the best-performing XGB model for emergency admissions. (c) Confusion matrix for clinician predicted and actual LoS quartiles for elective admissions. (d) Confusion matrix for clinician predicted and actual LoS quartiles for emergency admissions. (e) Confusion matrix for model predicted and actual LoS quartiles for elective admissions. (f) Confusion matrix for model predicted and actual LoS quartiles for emergency admissions. Q1, below 25% quantile (0–1 days for elective, 0–2 for emergency); Q2, 25% to 50% quantile (2–3 days for elective, 3–6 days for emergency); Q3, 50% to 75% quantile (4–10 days for elective, 7–14 days for emergency); and Q4, above 75% quantile (>10 days for elective, >14 days for emergency). Means with 95% CIs are calculated using bootstrap.

The macro-averaged F1 scores were 0.550 (model) versus 0.538 (clinicians) for elective and 0.422 (model) versus 0.308 (clinicians) for emergency admissions (figure 5a,b). The model was better at predicting those who were more likely to stay longer, reflecting that clinicians tended to be overly optimistic. For example, clinicians correctly predicted 70.0% and 56.0% of patients who were discharged in 0–2 or 3–6 days versus 64.5% and 28.8% from the model, while clinicians only correctly predicted 13.6% and 2.2% of patients who were discharged in 7–14 and >14 days versus 35.8% and 36.7% from the model (figure 5c–f).

## Discussion

Exploiting a wide range of features in EHRs, we developed machine learning models based on XGB, MLP and TabNet architectures to predict inpatient mortality and future LoS and explored whether a multitask learning strategy could further improve performance by predicting both outcomes simultaneously. Our models for predicting in-hospital mortality demonstrated strong predictive performance, with an AUROC of 0.92 for both elective and emergency admissions. For LoS prediction in clinically relevant quartiles, the best models achieved an AUROC of 0.83 and 0.72 for elective and emergency admissions, respectively. Notably, our models outperformed clinicians in predicting LoS for emergency patient, while providing similar performance in elective patients.

Various machine learning approaches have been used previously to predict in-hospital mortality among patients admitted as emergencies, with AUROCs ranging from 0.80 to 0.92.^7–9 34–36^ Our model is among the best reported for this task, and performance was stable when predictions were made at different times from admission. We achieved similar AUROC for both elective and emergency admissions.

Building on our earlier work predicting imminent hospital discharge within 24 hours, this study focused on longer LoS. Unlike most studies treating LoS as a continuous outcome or predicting prolonged LoS as a binary outcome,^15 37^ we categorised LoS into quartiles to provide more granular clinically relevant predictions for resource management. This approach aligns with recent research that reported AUROC values of 0.664 using structured data and 0.787 with a Large Language Model (LLM).^38^ We achieved better performance than their model using structured data for both admission types (AUROC: 0.833 (elective) and 0.716 (emergency)), and better performance than the LLM for elective admissions, potentially because we included much more detailed features. Our predictions were more accurate for elective than emergency admissions, reflecting more uncertainty in emergency admissions and greater requirements for social care post-discharge. We achieved better performance than a previous study predicting multiclass LoS for planned admissions^13^ (AUPRC 0.646 vs 0.556).

Comorbidity scores were the strongest predictors of mortality for both admission types. For elective admissions, laboratory test measurements were also critical, whereas emergency predictions relied more on age, body mass index, historical LoS and vital signs. For LoS prediction, the most influential features included hours since admission, hospital capacity factors, non-antibiotic medications and antibiotic use, reflecting clinical improvement and operational pressures. Age was a top predictor in emergency but not elective admissions.

Combined multiclass and multitask models potentially improve performance and reduce training time. However, overall, the performance improvements we observed were inconsistent and only modest when present. The multiclass model marginally improved LoS predictions for emergency admissions (AUROC: 0.726 (95% CI 0.725 to 0.727) vs 0.716 (95% CI 0.715 to 0.718)) but not for elective admissions. The multitask MLP model did not outperform the single-task MLP or XGB models, particularly for predicting LoS, possibly reflecting XGB’s superior handling of tabular data.^39 40^ Similar results have been reported in other multi-class classification problems^41 42^ and also for a multitask model that did not achieve better performance than individual models predicting mortality, acute kidney injury and reintubation.^21^ We also found that TabNet did not significantly outperform simpler models and required substantially longer training time, suggesting that performance was likely constrained more by the nature of input data than by complexity of model architecture. Therefore, XGB models trained within acceptable time periods demonstrated better predictive accuracy, making them more suitable for clinical applications. A few prior studies also reported multi-task learning for mortality and LoS predictions; however, they only focused on data from intensive care settings and relied on complex model architectures such as multimodal deep learning frameworks.^28 43^ In contrast, our study targets a much broader general hospital population including both elective and emergency admissions and uses routinely collected tabular EHR data that are widely available. By using interpretable and computationally efficient models such as XGBoost and MLP, our approach showed strong predictive performance, potentially improving feasibility and scalability for real-world deployment.

Our models matched or outperformed clinicians in predicting LoS, particularly for emergency admissions. Clinicians tended to overestimate early discharges, while our models provided more accurate predictions for longer stays. Furthermore, clinicians only predicted discharge dates sporadically in our setting (<5% of admissions) and even made non-sensical predictions of discharge dates in the past. Therefore, as well as potentially outperforming clinicians, our models also have the potential to deliver predictions consistently for all patients and at different timepoints throughout a hospital stay. Our results align with several previous studies showing similar or better performance from models than clinicians in predicting hospital discharges.^12 44^ From a clinical perspective, such automated prediction models could assist in discharge planning by alerting clinicians both to patients at risk of prolonged hospitalisation or deterioration and to readiness for discharge, facilitating discharge planning (including medication prescription) and reducing additional time spent in hospital waiting for discharge to occur. Accurate real-time estimates of LoS and mortality could also improve the efficiency of care delivery and patient recovery and optimal use of healthcare resources. To enable practical deployment, these models could be integrated into EHR systems and updated continuously as new data become available. Embedding model outputs into clinical dashboards or discharge planning tools, alongside uncertainty estimates or feature explanations, could support adoption while maintaining clinician oversight. However, challenges exist in clinical integration, such as conflicts with clinical autonomy, lack of trust in automated decision-support tools and restrictions in real-time data accessibility. Clear presentation of model outputs, interpretability and co-development with clinicians will be essential to support successful integration. Future research should explore real-time implementation in different hospital environments, evaluate the impact on clinical workflow and patient outcomes and ensure that models are interpretable and aligned with clinical reasoning. Further exploration into dynamic or continuously updated prediction models, and extension to other outcomes such as readmissions or intensive care unit admission, could help expand the utility of predictive models in healthcare systems.

Limitations of our study include that we did not consider multimodal data such as time-series, free text or radiology data. Using these data might improve performance but would require more complex model architectures, improved data quality and additional computational resources, potentially limiting applicability in some settings. We used data from the same hospital group for training and testing, but patient population, clinical workflow and documentation practices may vary across hospitals or institutions. Future work should perform external validation using data from hospitals in different regions or healthcare systems to strengthen the validity and generalisability of findings. Only a small proportion of admissions had clinician-predicted discharge dates recorded, and these were biased toward older patients with longer stays and higher mortality, which limits the generalisability of the comparison. We used ICD-10 diagnosis categories as features; these were recorded at discharge, but the information contained within them was likely known to clinicians and could be recorded in real-time. We also lacked data on hospital-level factors such as bed occupancy rates, which could influence predictions, particularly under operational pressure. Finally, predictions were made at a fixed time daily; however, our prior work showed the prediction time of day had limited impact on results.^29^

In conclusion, by integrating machine learning with comprehensive EHR data, our models achieved high performance in predicting in-hospital mortality and future LoS. The LoS model outperformed clinicians for emergency patients, with similar performance for elective patients, but provided predictions on 100% of patient-days in hospital versus just 3%–5% with clinicians. While combined multiclass XGB and multitask learning approaches offered some limited performance gains, single-task XGB models generally performed best and could be trained within acceptable time periods, making them well-suited for clinical implementation. Overall, our study demonstrates the potential of machine learning models to effectively manage hospital resources, improve patients’ recovery and facilitate healthcare delivery.

## Data Availability

Data may be obtained from a third party and are not publicly available. The datasets analysed during the current study are not publicly available as they contain personal data but are available from the Infections in Oxfordshire Research Database (https://oxfordbrc.nihr.ac.uk/research-themes/modernising-medical-microbiology-and-big-infection-diagnostics/iord-about/), subject to an application and research proposal meeting the ethical and governance requirements of the Database. For further details on how to apply for access to the data and a research proposal template, please email iord@ndm.ox.ac.uk. Code availability: a copy of the analysis code is available at https://github.com/jiaweioxford/ML_mortality_LoS.
